# Development of a nutrition and physical activity booklet to engage seniors

**DOI:** 10.1186/1756-0500-1-77

**Published:** 2008-09-04

**Authors:** Linda Burke, Peter Howat, Andy H Lee, Jonine Jancey, Deb Kerr, Trevor Shilton

**Affiliations:** 1 School of Public Health, Curtin University of Technology, GPO Box U 1987, Perth, WA 6845, Australia; 2 Centre for Behavioural Research in Cancer Control, Curtin University of Technology, GPO Box U 1987, Perth, WA 6845, Australia; 3 National Heart Foundation, Western Australia Division, 334 Rokeby Road, Subiaco, WA 6008, Australia

## Abstract

**Background:**

This paper describes the development and process evaluation of an interactive booklet aimed at motivating older adults to improve their nutrition and physical activity.

**Findings:**

The booklet was developed in consultation with seniors via focus groups, individual interviews and self administered questionnaires. The booklet was disseminated to a group of 114 seniors as the main component of a 12-week intervention program. Process evaluation was conducted during and at the end of the intervention period.

A large proportion of participants (86%) were engaged in the program in that they had, as a minimum, read the booklet. The majority of the participants found the booklet provided them with useful and interesting advice in an easy-to-read and informative manner. Three quarters (76%) reported the materials to be motivating and increased their awareness of nutrition and physical activity, while 79% intended to continue with changes to their physical activity and diet after the program concluded.

## Background

Seniors are a growing percentage of the worldwide population. For example, the number of people aged over 65 years in the United Kingdom is expected to rise to 20% of the total population by 2021 [1]. Similarly, about a quarter of the Australian population will be over 65 years old by 2021 [2]. Strategies need to be identified that will engage older adults in programs to control their increasing rates of diabetes, cancers, cardiovascular diseases and mental health problems [3-5].

Obesity is increasing among elderly people especially in industrialised countries [6]. Studies in Sweden and the USA showed that the prevalence of obesity increased by about 10% in less that a decade [4,6]. In Australia, more than 20% of people aged over 55 years are now obese, which puts them at higher risk for chronic diseases [7].

The rise in obesity levels is substantially due to a decreased energy expenditure in overall activity [3]. Participation in physical activity tends to decrease as people age [8]. Increase in physical activity can benefit a number of health outcomes such as heart disease, diabetes, some cancers, depression, osteoporosis and fall related injuries [3], as well as possibly reducing disability by up to five years and enhancing the quality of life [3,9,10].

Worldwide diet trends have shifted towards an increased energy intake (including fat and sugar). Fat levels consumed are now above the World Health Organisation's (WHO) recommended limit of 30% of daily energy intake. In the USA, only 35% of women and 39% of men aged over 60 years meet the fruit recommendations of two servings per day, while a mere 6% of men and women aged 60 years meet the vegetable recommendations of three servings per day [11]. Despite these significant deficiencies, many older people do not understand the need to change their diet [1].

Older people have different nutritional requirements from those of younger adults, yet few nutritional education programs have been specifically aimed at seniors [12]. Authorities recommend that more studies are needed to identify the determinants of healthy eating for seniors to improve the effectiveness of interventions[13].

Sedentary seniors are a difficult group to target and researchers have identified a need for alternative programs to face-to-face approaches to be trialled and evaluated. These alternative approaches include program delivery via telephone, internet, email, and post. The use of telephone with mailed intervention has been successfully used for maintenance of physical activity [14].

There is some evidence that a booklet on healthy eating and physical activity for sedentary seniors can encourage goal setting to improve these behaviours [1]. Specific health contracts written collaboratively by health professionals and seniors had positive impacts on exercise and physical activity behaviours [15].

When developing interventions for seniors, their special characteristics must be considered [16]. Including them in the program development and embracing their perceptions and experiences are likely to improve the success of interventions [17].

In this study, the Physical Activity and Nutrition for Seniors (PANS) program was developed based on a participatory action research (PAR) approach, in which a mailed booklet supported by telephone calls formed the main intervention. The objective of this paper is to describe the development and process evaluation of the interactive booklet.

## Findings

The development of the PANS intervention was based on a PAR approach involving systematic investigation and collaboration with the target group [18]. The process helps ensure that health promotion interventions are more relevant to the target group's needs. In the PANS program, the seniors were engaged in the development of the intervention throughout the whole process from early formative research, discussing the intervention type and its implementation, commenting on the proposed intervention, testing it and finally participating in post program evaluation.

### PANS intervention

PANS was a 12-week program aimed to improve the nutrition and physical activity levels of seniors through an interactive booklet, which contained advice and suggestions on how to set healthy goals. The intervention group was also provided with telephone support and motivational interviewing [19,20]. Process evaluation was conducted during and at the end of the intervention, and the relevant data are presented in this paper. A survey was also conducted at both pre- and post-intervention to evaluate behavioural changes which are reported elsewhere [21].

**Figure 1:**
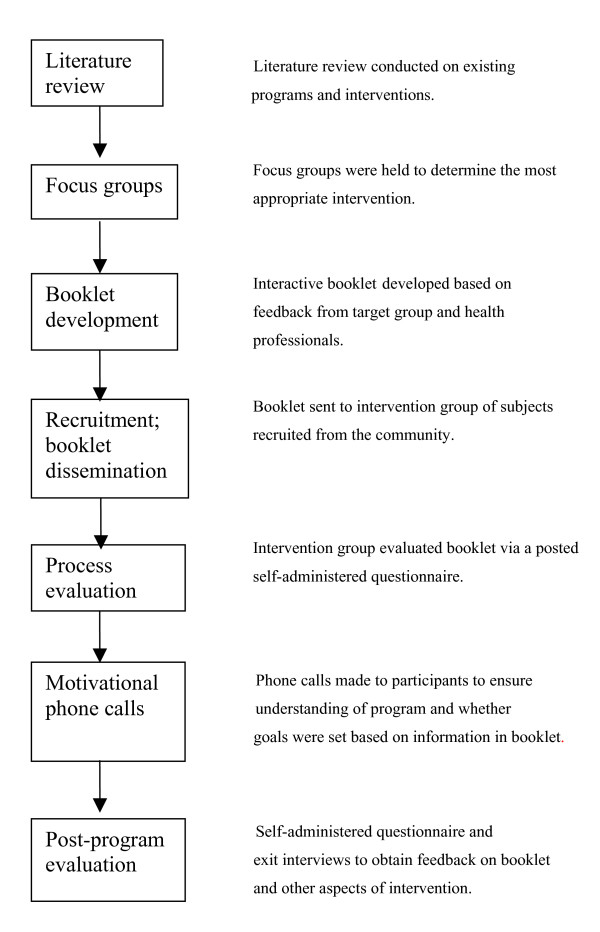
Process to develop and evaluate the intervention.

Figure 1 summarizes the process adopted to develop and evaluate the PANS intervention, and to illustrate the central role played by the mailed booklet.

#### Literature review

A literature review on nutrition and physical activity programs for seniors indicated a written intervention would be the most appropriate strategy. Evidence indicated that the intervention should be tailored to the seniors and be interactive to actively engage them. Advice in the form of an interactive booklet appeared to be most appropriate [22].

#### Focus group interviews

**Figure 2:**
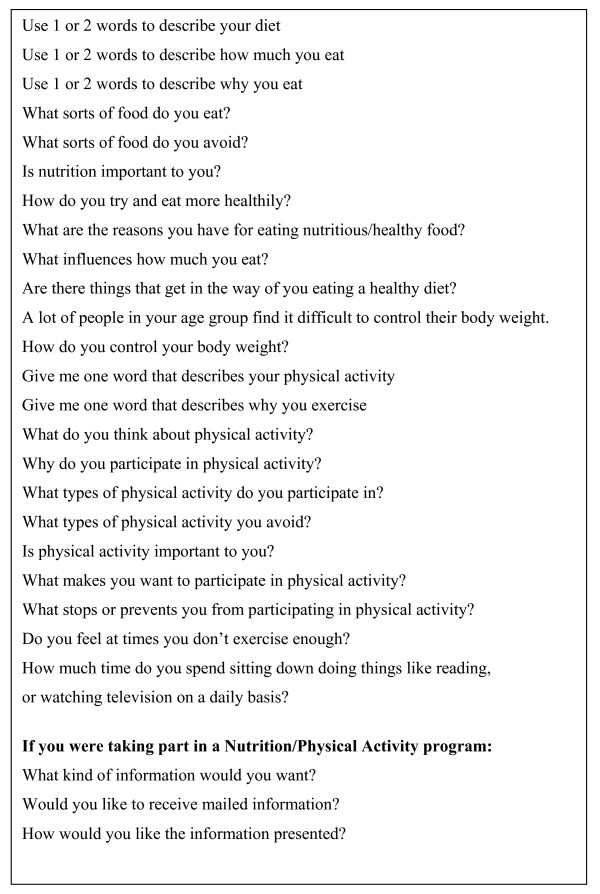
Focus group questions.

Four focus groups involving 40 seniors were conducted as part of the PAR approach. These focus groups gathered information on their nutrition and physical activity knowledge, attitudes and behaviours, along with suggestions for interventions suitable for their age group (Figure 2).

The responses indicated a desire for an interactive program that would enable the seniors to set their *own *goals based on their *individual needs*. Moreover, the participants liked the idea of receiving mailed information that was specifically addressed to them. A booklet was the most common format the target group agreed upon: "an attractive booklet would be good with large typeface print", as they thought single sheets of paper would get lost or discarded too readily. They were also receptive to receiving a few phone calls over a three month period (to support the booklet) as long as the calls were made at a pre-arranged time.

#### The booklet

A review of existing materials addressing nutrition and physical activity found them to be too long or complex. Development of a new booklet was informed by the advice gained from the focus group participants and from dieticians and physical activity specialists. It was deemed that a booklet which required the participants to write in would be useful and it should include information on: medical conditions, The Dietary Guidelines for Older Australians [23], the benefits of a healthy diet and physical activity guidelines along with goal setting. Special care was taken to ensure content was up to date, easy to read and not too overwhelming for the seniors. The final draft was pre-tested with a group of 20 seniors by way of self administered questionnaires and individual interviews [24]. Likert scale responses were used to assess their comprehension and acceptability of the presented messages, and other elements such as font size and illustrations, as recommended by the U.S. Department of Health and Human Services [25]. A pre-test of the final draft was also undertaken with the project staff to confirm the most effective presentation of the information.

**Figure 3:**
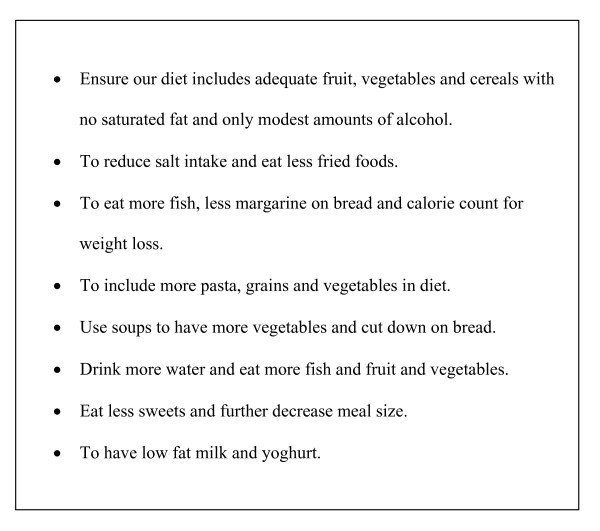
Examples of nutritional goals.

**Figure 4:**
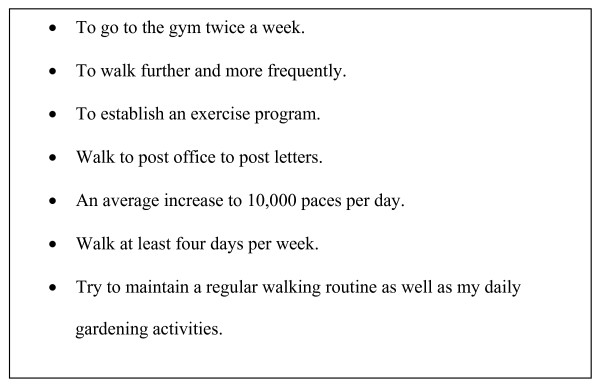
Examples of physical activity goals.

The booklet encouraged the seniors to set nutrition and physical activity goals in line with national recommendations. It explained health benefits of adopting good nutrition and participating in regular physical activity and gave clear guidelines on these. The final version of the booklet consisted of three sections. The first section introduced the program and asked questions about health concerns and issues based on the Health Belief Model. It explained how to address these health concerns and provided contact phone numbers and websites for specific health agencies. The second section gave information and examples on how to follow the Dietary Guidelines for Older Australians [23]. The seniors were then asked to set a couple of nutritional goals based on their own eating habits. Examples of nutritional goals are shown in Figure 3. The third section focused on physical activity. The national physical activity guidelines were discussed, with explanations on how the recommended 30 minutes of physical activity per day could be achieved. Benefits of participating regularly in physical activity were outlined and guidelines on how to start and plan physical activity were also addressed. [see Additional file 1]. The seniors were asked to set a couple of physical activity goals for the duration of the PANS program; see Figure 4. A table was presented at the end of the booklet to record the daily number of steps taken from a pedometer. A pedometer was given to each participant to encourage walking.

#### Recruitment and booklet dissemination

The program participants were randomly selected from the Australian Federal Electoral roll who resided within metropolitan Perth, the capital of Western Australia. Their names were matched to the Perth Electronic White Pages of telephone numbers. On initial phone contact prospective subjects were screened. Specifically, they were required to be aged 65–74 years and were excluded if they were considered too active or unhealthy. A total of 114 subjects (response rate 67% of eligible subjects contacted) were recruited into the intervention group.

All PANS participants were sent the booklet which was accompanied by a brief questionnaire. The questionnaire invited the seniors to rate specific features of the booklet such as: whether it was interesting, eye catching/attractive, easy to understand, if it contained useful advice, its suitability for the age group, and the relevance of messages. They were also asked to comment on anything they particularly liked or disliked as well as suggestions for improvement [25].

##### Motivational phone calls

Five weeks following the dissemination of the booklet, the PANS participants were phoned to check on their goal setting progress and to get feedback on their use of the booklet. There was also an opportunity to raise queries about the program.

### Program evaluation

Using a self administered questionnaire the participants were asked to describe significant changes in their health and fitness as a result of taking part in the 12 week intervention. Exit telephone interviews with a sample of the participants (n = 16) were also conducted at the conclusion of the PANS program. They were asked what aspects of the program they liked and disliked, and whether improvements could be made.

## Results

A large proportion of the participants (86%) returned the questionnaire that rated specific features of the booklet. Results, which were positive, are summarized in Table 1. The majority of seniors found the booklet interesting, agreed that it contained useful advice and that it encouraged them to think about their level of physical activity and nutrition. Qualitative responses included: "I found the suggested mix of foods interesting and handy to have as an ongoing reference"; "The portions of food was interesting, I will have to get my physical activity to a better level"; "The booklet prompted me to use it for myself and my husband"; "It made me look at what I had become slack on"; "The booklet gave me a greater understanding about physical activity"; "It reminded me of certain foods I need to eat".

**Table 1:** Booklet feedback results

**Comment**	**% Agree**
Useful advice in booklet	98
Suitability for age group	98
Interesting information in booklet	96
Attractive booklet	94
Easy to understand	95
Encouraged me to think about nutrition/physical activity	99

(n = 90)

Responses to the post-program evaluation survey were also very positive overall. Common responses included feeling fitter, having more energy and being more aware of their own health and fitness. As illustrated by the results of the survey in Table 2, the seniors found the program and materials motivating and appropriate. A majority of them (86%) claimed to have read the entire booklet. They did use the information provided to set nutritional and physical activity goals to suit individual needs.

**Table 2:** Post-program evaluation results

**Responses**	**%**
Read through the booklet	86
Booklet feedback form did encourage me to read the booklet	70
Found the program and materials motivating	76
Did not take a long time to read the booklet	72
Feel healthier since starting the program	54
Became more aware of health and well-being	76
More likely to do something about my health and well-being	64
Walk more often	57
Am generally more active	57
Could get more done in a day	49
Will continue to be more active when the program concludes	78
Will be more active in 6 months time	58
Will be more active in 12 months time	51
Will continue to maintain a healthy diet when program concludes	78
Will still maintain a healthy diet in 6 months time	79
Will still maintain a healthy diet in 12 months time	78
Had set some nutritional goals	43
Had reached some of these nutritional goals over the last 12 weeks	42
Had set some physical activity goals	35
Had reached some of these physical activity goals over the last 12 weeks	33
Became involved in new activities	25
Have changed diet since starting program	41

(n = 114)

**Figure 5:**
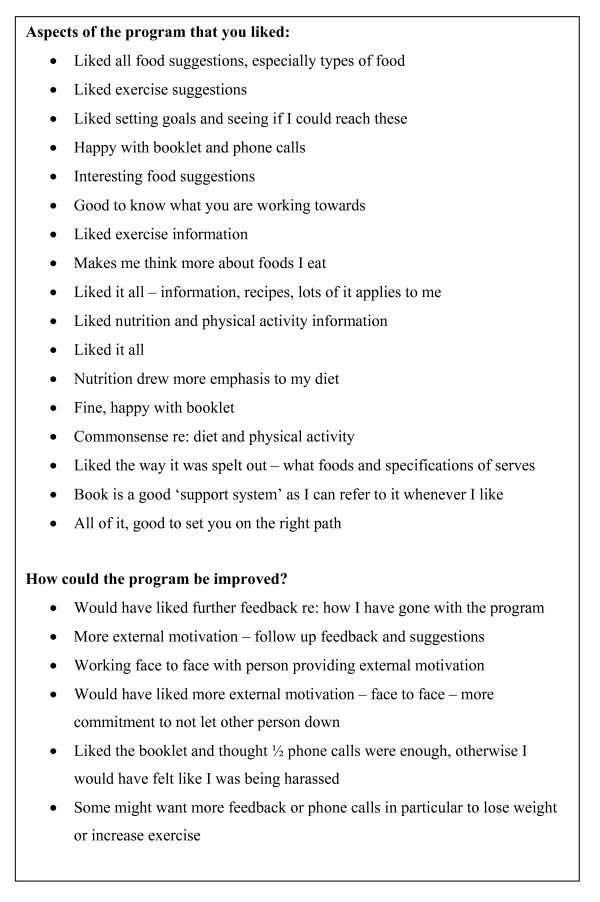
Feedback from exit interviews (n = 16).

Feedback from the exit interviews indicated that seniors were positive about the booklet (Figure 5).

## Discussion and conclusion

That many seniors are physically inactive and overweight is a compelling reason for the development of physical activity and nutrition interventions tailored to their specific circumstances [4-8]. The challenge is to get this target group exposed to a sufficient dose of an intervention that will induce and maintain behaviour change [9].

The literature has reported many programs that improve physical activity levels of participating seniors. But most of these involve group participation or attendance at recreation or other venues. While there has been an increase in home-based physical activity programs for younger age groups, few are reported for seniors [14,19]. This is particularly so for combined physical activity and nutrition programs [1,11,12,20]. It has been suggested that booklets can be used effectively for people older than 65 years in order to encourage them to set goals for increased participation in physical activity and improved diets [1].

The steps undertaken as described in this paper provide guidelines useful for ensuring health promotion materials developed for seniors are relevant [9,12]. This process also helps ensure that the target group will receive a significant dose of the intervention, and thus increases their chances of behaviour change.

## Competing interests

The authors declare that they have no competing interests.

## Authors' contributions

LB conducted the PANS program and drafted the manuscript. PH designed the study, coordinated the project and revised the manuscript. AL performed data analysis and revised the manuscript. JJ participated in the study design and program evaluation. DK and TS provided expert advice and supported the development of the intervention. All authors read and approved the final manuscript.

## Supplementary Material

Additional file 1 A copy of the booklet sent to all intervention group participants. Click here for file
