# Supplementary material for: Development of a nutrition and physical activity booklet to engage seniors
Source: BMC Res Notes. 2008 Sep 4;1:77. doi: 10.1186/1756-0500-1-77 (PMC2547105; doi:10.1186/1756-0500-1-77)

**Perth Active Living for Seniors (PALS)**

Program

**Improving quality of life through physical activity and nutrition**


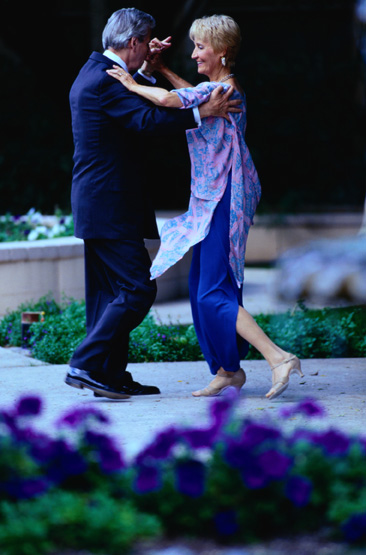


*“The most serious problem emerging in Australian Society is obesity...It is up to us older group, not only to be active for our own health benefit, but importantly to set an example to our kids and their kids. If we don't do it they won't listen”*

Herb Elliot (1960 Olympic champion), March 2006.

**A project of the Western Australian Centre for Health Promotion Research, Curtin University,**

**the National Heart Foundation of Australia (WA Division)**

**in collaboration with the Australian Technology Network Universities Centre for Metabolic Fitness**

**2006**

*Acknowledgements*

*The ATN Centre for Metabolic Fitness and Healthway*

*This booklet was developed by: L Burke, A Clarke, P Howat, J Jancey,*

*K Kennington, D Kerr, A Lee, C Pollard, T Shilton and S White.*

*Photographs were used with the permission of the Centre for Research into Aged Care Services (CRACS) WA*

**Perth Active Living for Seniors (PALS)**

Program

The goal of the PALS program is to assist you to promote and maintain your health through healthy eating and being active. It is not always easy to know what to eat or how to find time to be active.

Use this booklet to help you assess your nutrition and physical activity habits. For those wanting to make changes, it will guide you to achieving your goals over the10-weeks.


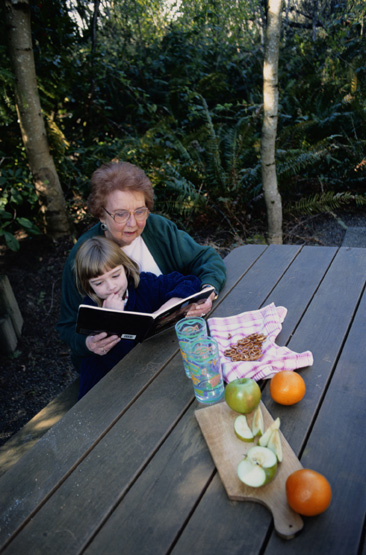


You will receive a couple of phone calls (at times convenient to you) to check if you need any more information or support.

**Contacts**

If you have any queries call Linda Burke on 9266 7997 (Wednesdays and Fridays 10am to 2pm) or email [Linda.Burke@exchange.curtin.edu.au](mailto:Linda.Burke@exchange.curtin.edu.au)

**Health concerns and issues**

Think about your health now. Are any of the following concerning you? Circle and rate your level of concern.

**0= not concerned 10= concerns me a lot**

**1. Heart disease/Stroke**

0 1 2 3 4 5 6 7 8 9 10

**2. Diabetes**

0 1 2 3 4 5 6 7 8 9 10

**3. Cancer**

0 1 2 3 4 5 6 7 8 9 10

**4. Osteoporosis**

0 1 2 3 4 5 6 7 8 9 10

**5. Arthritis**

0 1 2 3 4 5 6 7 8 9 10

**6. High blood pressure**

0 1 2 3 4 5 6 7 8 9 10

**7. High cholesterol**

0 1 2 3 4 5 6 7 8 9 10

**8. Constipation**

0 1 2 3 4 5 6 7 8 9 10

**9. Being overweight**

0 1 2 3 4 5 6 7 8 9 10

**10. Being underweight**

0 1 2 3 4 5 6 7 8 9 10

**Did you know that all of the above health issues can be managed with good nutrition and regular physical activity?**

**Managing health issues with good nutrition and regular physical activity**

**1. Heart disease/Stroke**

Maintaining a healthy weight, keeping physically active, reducing saturated (animal) fat and eating a nutritious diet helps.

Try reducing:

- meat serving sizes (90-120g is ideal)
- butter, dripping and lard

Try removing:

- fat on meat & chicken skin before cooking

Try eating more:

- fruits and vegetables
- legumes (lentils, chick peas, baked beans)
- fish meals (three times a week is ideal)

Contact the Heart Foundation on 9388 3343 or visit the website [www.heartfoundation.com.au](http://www.heartfoundation.com.au/)

**2. Diabetes**

Maintaining a healthy weight, keeping physically active and eating a nutritious diet can help prevent and manage diabetes.

Contact the Diabetes Australia-WA on 9325 7699 or visit the website [www.dawa.com.au](http://www.dawa.com.au/)

**3. Cancer**

Maintaining a healthy weight, keeping physically active and eating a nutritious diet (with plenty of fruit and vegetables) and limiting alcohol intake can reduce the risk of developing cancer.

Try reducing alcohol intake by:

- having alcohol free days
- drinking non-alcoholic drinks

Contact the Cancer Council Western Australian on 9212 4333 or visit the website [www.cancer.com.au](http://www.cancer.com.au/)

**4. Osteoporosis**

Keeping physically active (especially bone-loading exercise) and eating a nutritious diet (with plenty of calcium-rich foods) can help.

Try increasing calcium intake:

- eat dairy foods (milk, cheese or yoghurt).
- choose lower fat dairy foods (e.g. reduced fat or skim milk) as these have extra calcium and less saturate fat.
- choose soy milk enriched with calcium

Try bone-loading exercise:

- gym-based exercise with weights
- weight-bearing exercise (e.g. walking, tennis and dancing)

Contact the Osteoporosis Western Australia 9388 2199 or visit the website [www.osteoporosis.org.au](http://www.osteoporosis.org.au/)

**5. Arthritis**

There are many forms of arthritis but the most common is osteoarthritis (OA). Maintaining a healthy weight, keeping physically active and eating a nutritious diet can help with OA.

Try increasing:

- fish meals (especially oily fish e.g. salmon, sardines) to three times a week

Contact the Arthritis and Osteoporosis Foundation or visit the website [www.arthritisaustralia.com.au](http://www.arthritisaustralia.com.au/)

**6. High blood pressure**

If you have high blood pressure, losing weight (if you are overweight), increasing physical activity, reducing alcohol consumption and having less salt could all help.

Contact the Heart Foundation on 9388 3343 or visit the website for more information [www.heartfoundation.com.au](http://www.heartfoundation.com.au/)

**7. High Cholesterol**

Maintaining a healthy weight, keeping physically active, reducing saturated (animal) fat and eating a nutritious diet helps.

Try reducing:

● meat serving sizes (90-120g is ideal)

● butter, dripping and lard

● take-away foods to once a week (e.g.

pies, pizza, hamburgers)

Try removing:

● fat on meat & chicken skin before cooking

Try eating more:

● fruits and vegetables

● legumes (lentils, chick peas, baked

beans)

● fish meals (three times a week is ideal)

Try using:

● a variety of oils when cooking (e.g. olive

oil, canola, sunflower, soybean)

Contact the Heart Foundation on 9388 3343 or visit the website [www.heartfoundation.com.au](http://www.heartfoundation.com.au/)

**8. Constipation**

Increasing your dietary fibre intake, drinking more water and increasing physical activity can help with constipation. Some medications can make you more prone to constipation.

Try increasing fibre:

- Eat more fresh fruit, such as apples and pears (with the skin on), bananas.
- Choose wholemeal or multigrain bread
- Eat more salad and vegetables
- Add dried fruit (to cereal or snacks)

**9. Being overweight**

Weight gain occurs when your food intake (kilojoules) exceeds how much energy you burn with activity. Before trying to lose weight discuss with your doctor. Lose weight sensibly, stay away from fad diets, choose nutritious foods, eat a little less and be more active.

Some tips to losing weight:

- Examine your food portions. Are you overdoing some foods? Check portion sizes against the Australian Guide to Health Eating.
- Re-arrange your plate -increase the serves of vegetables and limit meat to about 90 -120g.
- Limit how much fat you eat; e.g. fried foods, fatty meat, butter on bread.
- Cut down the amount of alcohol your drink to less than two standard drinks (e.g. a middy of beer, a nip of spirits or a 150ml glass of wine) a day and limit the number of days you drink.
- Increase physical activity and energy expenditure.

**10. Being underweight**

Maintaining a healthy body weight can be difficult if your appetite is not great. Breaking your food intake up over the day can help. For example have a number of snacks during the day instead of three main meals. Increase your intake of milk and milk drinks may also help. Any unintentional weight loss should be discussed with your doctor.

**Nutrition increases vitality and Energy**

**Good nutrition** is important, as its central to maintaining quality of life and independence.

**How am I doing?**

Take a minute to think about how you eat. Ask yourself, how important is it to you to eat a healthy diet? *If 0 is ‘not at all important’ and 10 is ‘very important’ what number would you give yourself?* Circle the number below

0 1 2 3 4 5 6 7 8 9 10

If you decided right now to change how you eat, how motivated or interested do you feel about succeeding with this? If 0 is ‘not at all motivated’ and 10 is ‘very motivated’ what number would you give yourself?

0 1 2 3 4 5 6 7 8 9 10

If you scored less than ‘8’, you could probably use a little help with motivation. Write down what it would take to increase your motivation up to a 9 or 10

________________________________________________________________________________________________________________________________________________________________________________

**How does the food you eat rate?**

Dietary Guidelines for Older Australians are designed to assist you to promote and maintain your health and wellbeing.

They are:

- Eat a wide variety of nutritious foods.
- Eat at least three meals every day.
- Eat plenty of vegetables (including legumes) and fruit every day.
- Eat a diet low in saturated fat.
- Include foods high in calcium.
- Choose foods low in salt and use salt sparingly.
- Drink adequate amounts of water and/or other fluid.
- Keep active to maintain muscle strength and a healthy body weight.

**Eat a wide variety of nutritious foods**

Choose foods from each of the 5 food groups. Make sure you choose a variety of foods from within each food group. For example, within the vegetable group choose a variety of dark green and yellow vegetables (raw and cooked)

**The 5 food groups are:**

1. Bread, cereals, rice, pasta and noodles
2. Vegetables including legumes
3. Fruit
4. Milk, yoghurt and cheese
5. Meat, fish, chicken, eggs, nuts and legumes

**
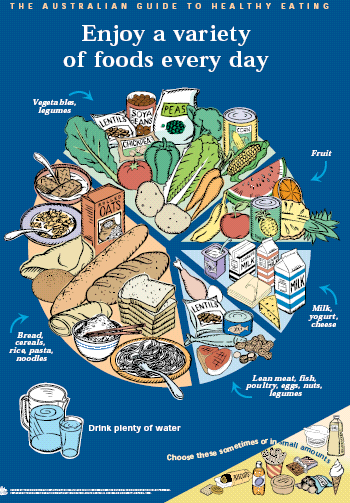
**

**Eat at least three meals every day**

You need to eat enough food to provide energy and good nutrition. As you get older your appetite may reduced. Some people are at risk of being **underweight**. Missing meals means you could be missing out on good nutrition.

Here are some quick and easy healthy eating tips:

#### *Breakfast*

Fresh fruit, cereal, porridge and milk

***Lunch***

Soup (e.g. split pea)

Sandwich or bread roll (e.g. cold meat, canned fish, cheese and salad)

Yoghurt

***Snack***

Fruit

***Dinner***

Lean meat, fish, chicken

Vegetables or salad

Rice, pasta or potatoes

Fruit with custard

**Eat plenty of vegetables per day (including legumes)**

‘Eating plenty’ means eating at least five serves of vegetables and two serves of fruit each day. This is not as hard to achieve as you might think!

**What are legumes?**

Legumes are beans (e.g. split peas, baked beans, lentils, kidney and soy beans). They are a good source of fibre and low in fat.

**What is a serve of vegetables?**

▪ 1 cup of salad

▪ 1 medium potato

▪ ½ a cup of cooked vegetables

▪ ½ a cup of cooked, dried or canned

beans, peas or lentils


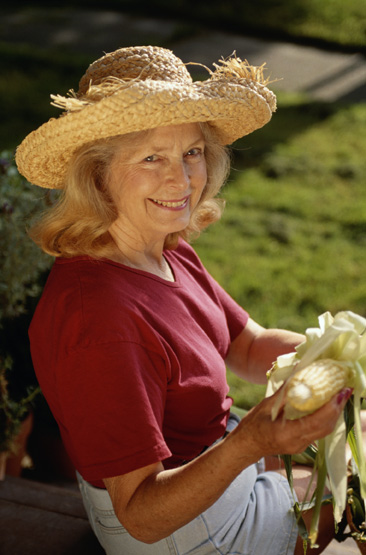


Making a vegetable soup with lentils, potatoes, carrots, pumpkin and celery gives you two to three vegetable servings for the day.

**What is a serve of fruit?**

Try eating **two servings** of fruit every day (fresh, frozen, canned, or dried). Fruit juice contains vitamins but lacks fibre.

**One serve equals:**

▪ 1 medium piece (an apple, banana,

orange or pear)

▪ 2 small pieces (apricots, plums or

kiwi fruit)

▪ 1 cup diced pieces or canned fruit

▪ ½ cup 100% juice

▪ 1½ tablespoons dried sultanas or 4

dried apricots


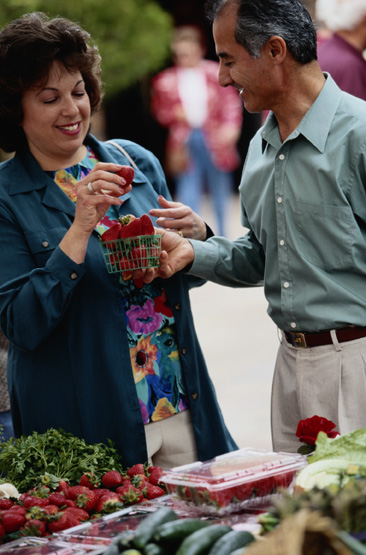


Ideas to get extra fruit serves:

- Chop up some fresh, dried or canned fruit and add to your cereal or as a dessert.
- Freeze any overripe bananas and use them to make a milkshake.
- Stew or bake fruit
- Make a banana custard
- Make fruit your snack

**Eat a diet low in saturated fat**

Too much saturated fat can increase your risk of heart disease. To reduce your saturated fat (animal fat) intake:

- Avoid fatty meats and cut off any visible fat before cooking.
- Use low fat cooking methods such as grilling, dry baking or steaming.
- Avoid using dripping or lard as these are high in saturated fats
- Avoid fried foods
- Choose low fat dairy foods
- Have fish (canned or fresh) twice a week
- Bake your own cakes and biscuits using polyunsaturated or monounsaturated margarine or oil - commercial cakes and biscuits can be high in saturated fat.
- Use polyunsaturated or monounsaturated (olive) oil instead of butter in cooking.

Choose foods that are low in saturated fat. Read the label to check its low in animal and has a total fat content below 10g per 100g serve.

**Include foods high in calcium**

Calcium is important for your bone health and can help protect against Osteoporosis. This is a condition where your bones become fragile and fracture easily. As you get older it is especially important you keep up your calcium.

Try to have three to four servings of milk or milk products every day, as these are excellent sources of calcium.

One serve equals:

- 1 glass of milk or calcium-fortified soy milk
- 1 small tub of yoghurt
- 1 cup custard
- 40 grams of cheese (2 slices)

**Choose foods low in salt and use salt sparingly.**

Salt or sodium chloride occurs naturally in many foods, added during food production or at the table. To reduce your salt intake:

- Try not to add salt to your food at the table
- Choose low-salt foods where available
- Use pepper, fresh herbs and spices instead of salt in cooking

**Drink adequate amounts of water and/or other fluid**

Our bodies need water to function properly and prevent constipation. Not drinking enough water can lead to dehydration. *(Department of Health WA 2005)*

Drink at least 6 to 8 glasses of fluid per/day.

Drink water regularly throughout the day, especially if you are active or gardening outside.

Try these ideas:

▪ Keep water chilled and serve it in a special

glass or bottle.

▪ Add frozen pieces of fruit to water

▪ Add lemon slices to give the water a

refreshing taste

Fluids may also include soup, coffee, tea, juice, custard, milk (although coffee and tea have a mild diuretic effect)

*(Eat Well For Life 2000).*

**Keep active to maintain muscle strength and a healthy body weight**

Being physical active is important helping you maintain a healthy body weight and muscle strength. See page 20 to assess your physical activity level.

**Setting nutrition goals**

*How did you go? Now that you have looked at what you eat is there something you would like to change about how you are currently eating? Setting a specific goal is a great way to go about changing what you eat.*

*Here are some examples of goals you may wish to try:*

- *I plan to eat 2 serves of fruit every day*
- *To increase my vegetable intake I will add salad to my sandwich at lunch*
- *To cut down on saturated fat in my diet I will take the skin off the chicken and cut the fat off meat before cooking*

If you would like to set some eating goals, write these down in the space below.

**My** goals

1. ____________________________________

2. ____________________________________

3. ____________________________________

*Please answer the questions on the following page in order to help you assess your physical activity*

**1. Participation in moderate physical activity**

**
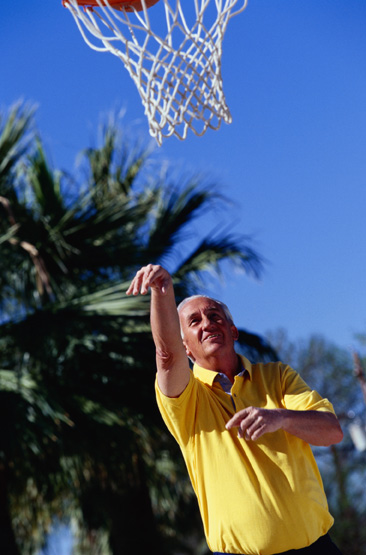
**

Days per week _____

Average number of minutes

per day ______

# 2. Walking for physical activity

Days per week, for at least 10 minutes _____

Average number of minutes per day when you

walk _____

#

# 3. Spend less time sitting down

# Average number of minutes per day spent doing sitting activities such as: watching television or using the computer_____

*Compare your answers to the information on the following pages to see if* ***your*** *level of physical activity can be improved.*

**How does your physical activity rate?**

*National Guidelines state that for good health, adults should do at least 30 minutes of moderate-intensity physical activity on all or most days, of the week.*

Moderate activity is when you notice a visible increase in the rate and depth of your breathing, but you can still have a conversation e.g. brisk walking.

*The Heart Foundation recommends you aim for 10,000 steps per/day in order to achieve good health.*


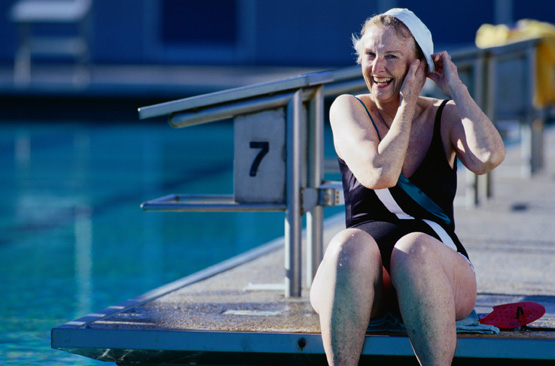
If you are currently doing less, aim to increase this by 2,000 to 3,000 steps per/day by the end of this program. This amount of walking would increase your daily physical activity by 30 minutes.

**What counts as physical activity?**

*The National Heart Foundation defines physical activity as “any activity you do which involves the large skeletal muscles”.*

This includes activities such as walking, swimming, dancing, golf, cycling, gardening, tai chi, jogging and any sport.

**Aim for 30 minutes of moderate-intensity physical activity every day**

This activity can be accumulated in bouts of 10 minutes or more if that is more convenient. You should aim to do 30 minutes on most days of the week. However it is more beneficial to your health to be active every day of the week.

At any age, physical activity provides a range of health benefits.

Most adults don’t need to visit their doctor before commencing some moderate physical activity. However, if you have a chronic condition such as heart disease or diabetes, **it is advisable to consult your doctor first**.

**Physical Activity**

**Those who participate in regular physical activity tend to:**

- **Have improved long term health**
- **Have a reduced risk of heart disease, stroke, diabetes and certain cancers.**
- **Manage their weight better**
- **Have healthier blood cholesterol levels**

**and lower blood pressure**

- **Have stronger bones and muscles (less osteoporosis and less risk of having a fall)**
- **Recover better from a heart attack (if this has occurred)**
- **Feel more energetic, confident, happy, relaxed and are able to sleep better**
- **Have improved heart and lung function**
- **Improved posture, endurance, joint mobility, balance and strength**
-
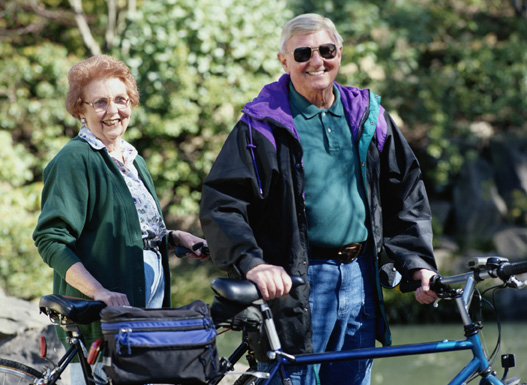
**Better leg circulation, reducing the risk of varicose veins**

**Intensity**

Don’t over do it, try the “Talk Test”

Make sure you can talk without puffing. However, try not to dawdle if chatting, and keep your walking pace fast enough to get your pulse (heart rate) up without making yourself breathless or uncomfortable.

**Start where you are**

At a level comfortable to you and gently increase over time.

If you are not up to a 30-minute walk, try to make it add up to 30 minutes, in smaller pieces. Add duration in five-minute increments. Be sure it is gradual.

**Preparation**

Wear comfortable shoes; buy a shoe that feels like a bedroom slipper.


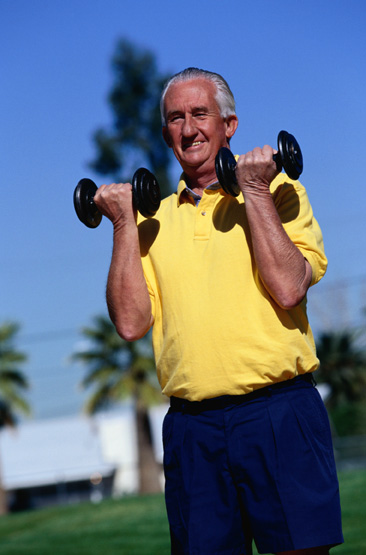
 *(S.H.A.P.E. project)*

**Support**

Do it with one friend or more, even join a group.

**Make it a habit**

Mark down the days and times in your calendar, so you don’t double book and miss out. ***Enjoy it!***

**Setting physical activity goals**

*How did you go? Now that you have looked at your level of physical activity is there something you would like to change about it? Experts have found that setting a specific goal is a great way to go about changing your level of physical activity.*

*Here are some examples of goals you may wish to try:*

- *I plan to increase the number of steps I take by 2000 per day by the end of this program*
- *I will try and walk when taking short trips in my neighbourhood instead of driving*
- *I plan to exercise with a friend to increase my level of physical activity*

If you would like to set some physical activity goals, write these down in the space below.

**My** goal is to

1. ____________________________________

2. ____________________________________

3. ____________________________________

**Optional**

This page can be used to **record** the number of steps you have taken **per day**, as recorded on your pedometer. *You may find that by keeping track of your steps on the table below it increases the number of steps you take per day.*

## Number of steps taken per day

| Week | Mon | Tue | Wed | Thu | Fri | Sat | Sun |
| --- | --- | --- | --- | --- | --- | --- | --- |
| 1 |  |  |  |  |  |  |  |
| 2 |  |  |  |  |  |  |  |
| 3 |  |  |  |  |  |  |  |
| 4 |  |  |  |  |  |  |  |
| 5 |  |  |  |  |  |  |  |
| 6 |  |  |  |  |  |  |  |
| 7 |  |  |  |  |  |  |  |
| 8 |  |  |  |  |  |  |  |
| 9 |  |  |  |  |  |  |  |
| 10 |  |  |  |  |  |  |  |

Remember to reset your pedometer at the end of each day after you have recorded the number of steps taken on the table above. You will then be able to see how you are improving by comparing the number of steps taken per day.

**A project of the Western Australian Centre for Health Promotion Research, Curtin University,**

**the National Heart Foundation of Australia (WA Division)**

**in collaboration with the Australian Technology Network Universities Centre for Metabolic Fitness**


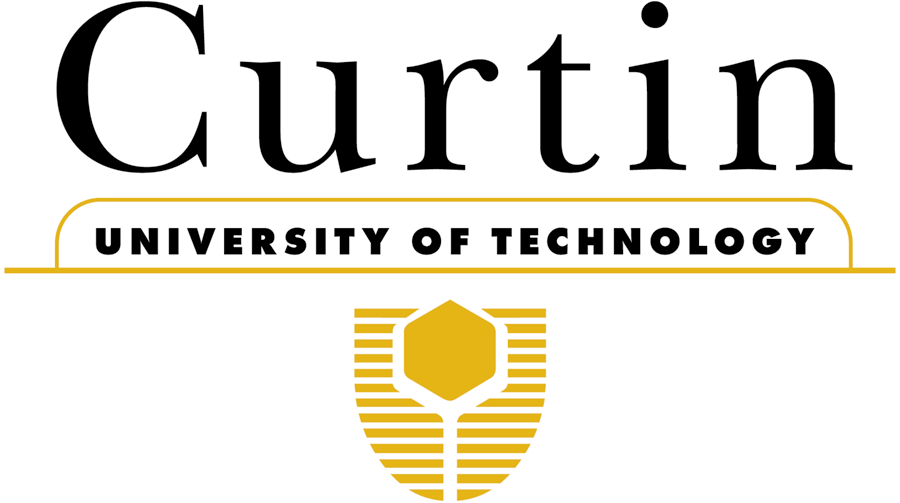
 **
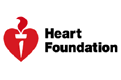
**

***References***

*Australian Nutrition Foundation Inc 2006*

*Department of Health and Aged Care WA 2005*

### Dental Health Education Unit 1998

### Eat Well For Life 2000

*National Health and Medical Research Council 1999 (NHMRC)*

*The Australian Guide to Healthy Eating 1998*

*S.H.A.P.E. project Oregon Research Institute*


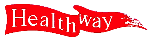

Supplement: Additional file 1 — A copy of the booklet sent to all intervention group participants. [file 1756-0500-1-77-S1.doc]
